# Academic Physician and Trainee Occupational Well-Being by Sexual and Gender Minority Status

**DOI:** 10.1001/jamanetworkopen.2024.43937

**Published:** 2024-11-13

**Authors:** Carl G. Streed, May Navarra, Jessica Halem, Miriam T. Stewart, Susannah G. Rowe

**Affiliations:** 1 Section of General Internal Medicine, Department of Medicine, Boston University Chobanian and Avedisian School of Medicine, Boston, Massachusetts; 2 GenderCare Center, Boston Medical Center, Boston, Massachusetts; 3 Eidos Initiative, University of Pennsylvania School of Nursing, Philadelphia; 4 Division of General Pediatrics, Children’s Hospital of Philadelphia, Philadelphia; 5 Department of Pediatrics, Perelman school of Medicine, University of Pennsylvania, Philadelphia; 6 Office of Equity, Vitality and Inclusion, Boston University Medical Group, Boston Medical Center and Boston University Chobanian and Avedisian School of Medicine, Boston, Massachusetts; 7 Department of Ophthalmology, Boston University Chobanian and Avedisian School of Medicine, Boston, Massachusetts

## Abstract

**Importance:**

Few studies have explored the association between sexual and gender minority (SGM) status and occupational well-being among health care workers.

**Objectives:**

To assess the prevalence of burnout, professional fulfillment, intent to leave, anxiety, and depression by self-reported SGM status.

**Design, Setting, and Participants:**

This cross-sectional survey study collected data from October 2019 to July 2021, from 8 academic medical institutions participating in the Healthcare Professional Well-Being Academic Consortium. The survey, including questions on SGM status, was administered to attending physicians and trainees. Statistical analyses were performed from June 1, 2023, to February 29, 2024.

**Exposure:**

SGM status was determined via self-reported sexual orientation and gender identity.

**Main Outcomes and Measures:**

Primary outcomes measured were the Professional Fulfillment Index (burnout and professional fulfillment), intent to leave, and self-reported anxiety and depression using the Patient-Reported Outcomes Measurement Information System short-form 4-item measure.

**Results:**

Of 20 541 attendings and 6900 trainees, 8376 attendings and 2564 trainees responded and provided SGM status. Of these respondents, 386 attendings (4.6%) and 212 trainees (8.3%) identified as SGM. Compared with their non-SGM peers, SGM attendings had a lower prevalence of professional fulfillment (133 of 386 [34.5%] vs 3200 of 7922 [40.4%]) and a higher prevalence of burnout (181 of 382 [47.4%] vs 2791 of 7883 [35.4%]) and intent to leave (125 of 376 [33.2%] vs 2433 of 7873 [30.9%]) (all *P* < .001). Compared with their non-SGM peers, SGM trainees had a lower prevalence of professional fulfillment (63 of 211 [29.9%] vs 833 of 2333 [35.7%]) and a higher prevalence of burnout (108 of 211 [51.2%] vs 954 of 2332 [40.9%]) (both *P* < .001). After adjusting for age and race and ethnicity, SGM attendings had higher odds of burnout than their non-SGM peers (adjusted odds ratio, 1.57 [95% CI, 1.27-1.94]; *P* < .001). Results for burnout were similar among the SGM trainees compared with their non-SGM peers (adjusted odds ratio, 1.47 [1.10-1.96]; *P* = .01).

**Conclusions and Relevance:**

In this cross-sectional survey study of academic physicians and trainees, SGM attendings and trainees had higher levels of burnout and lower levels of professional fulfillment. SGM attendings had greater intent to leave than their non-SGM peers, but trainees did not. These disparities represent an opportunity for further exploration to retain SGM health care workers.

## Key Points

**Question:**

What is the association of sexual orientation and gender minority (SGM) status with burnout, professional fulfillment, and intent to leave among academic physicians and trainees?

**Findings:**

In this cross-sectional survey study of 10 940 academic physicians and trainees, SGM status was associated with burnout and lack of professional fulfillment. SGM attendings expressed a higher intent to leave than their non-SGM peers, but SGM trainees did not.

**Meaning:**

These findings suggest that to be effective, interventions to address burnout, professional fulfillment, and retention should explicitly address disparities in occupational well-being experienced by SGM physicians and trainees.

## Teaser

This cross-sectional survey study uses a national survey to describe occupational well-being, including burnout, professional fulfillment, and intent to leave the profession, in health care settings among attending physicians and trainees who report sexual orientation and gender minority status.

## Introduction

Burnout is a public health crisis that affects the well-being of physicians and the populations they serve.^1,2^ Burnout is characterized by emotional exhaustion, cynicism, lack of motivation, and feelings of ineffectiveness and inadequate achievement at work.^3^ Compared with the general working US population, physicians are at increased risk for burnout and less likely to be satisfied with their work-life balance.^4^ Physician burnout and job dissatisfaction are associated with poorer functioning of health care institutions,^5^ increased intention to reduce work hours or leave their institution,^6,7,8^ reduced quality of care and poor patient outcomes,^8,9^ increased medical errors,^10,11^ and patient dissatisfaction.^12^

According to the American Medical Association, the COVID-19 pandemic exacerbated challenges for both patients and physicians.^13^ Increased work hours, dissatisfaction with work-life integration, childcare stress, limited resources, and concerns over transmitting the virus to family members were drivers of physician burnout.^13,14^ Across all measured demographics of physicians, pandemic-related burnout was high,^15^ and these higher rates of burnout have persisted as the acute phase of the pandemic has passed,^2^ accelerating physician shortages and plummeting access to quality health care.^16,17^ A step in addressing these challenges is to identify populations of physicians at greatest risk of burnout and of subsequently leaving the workforce.

Sexual and gender minority (SGM; ie, lesbian, gay, bisexual, transgender, and queer [LGBTQ]) clinicians experience unique workplace stressors. In a recent survey of anesthesiologists, SGM status was a strong independent risk factor for burnout.^18^ Similarly, data from the Association of American Medical Colleges revealed that medical students with minority sexual identity (ie, lesbian, gay, and bisexual [LGB]) were more likely to report perceived mistreatment and have greater likelihood of burnout compared with non-LGB students.^19^ Regardless of their sex or race and ethnicity, LGB students had higher exhaustion and disengagement scores.^20^

While some data on trainees are available, very little is known about burnout among SGM physicians, nor do we know what factors contribute to resiliency in this population.^19,21^ Focusing on retention of SGM physicians is critical to the quality of patient care, since having a physician workforce inclusive of SGM physicians has been shown to be associated with improved patient outcomes, reduced stigmatization of SGM patients, and enhanced workforce development.^22,23,24,25,26,27^ A better understanding of the occupational well-being of SGM clinicians will allow for identification, development, and dissemination of strategies at the individual and system level to make health care settings more welcoming and inclusive of SGM people, mitigate burnout among SGM clinicians, foster resilience and retention, and preserve access to care for all patients.^28^ This study aims to describe differences in occupational well-being measures for SGM vs non-SGM clinicians, using a national survey sampling physicians across specialties.

## Methods

### Study Design

A cross-sectional survey study of attending physicians and trainees in the US was performed at 8 health care organizations participating in the Healthcare Professional Well-Being Academic Consortium.^29^ As described in detail elsewhere,^17^ a validated survey to assess professional fulfillment, burnout, and intent to leave was administered among attending physicians and trainees between October 2019 and July 2021. Surveys were voluntary, and participants were permitted to skip questions they preferred not to answer or that did not apply. Surveys were streamlined to include the same core measures (ie, burnout, professional fulfillment, intent to leave, and demographics) and customized to allow individual sites to assess hypothesized proximal determinants of well-being that align with their unique program goals and action areas. Surveys were distributed via individual email to each participant with up to 6 reminders for individuals who had not yet completed the Professional Fulfillment Index (PFI), the survey’s primary outcome measure. An independent professional survey vendor conducted all surveys and analyses. The Stanford Human Subjects Review Board deemed this study exempt from approval and informed consent as it uses deidentified, previously collected data. This study followed the American Association for Public Opinion Research (AAPOR) reporting guideline.

### Data Coding and Preparation for Analysis

After the conclusion of data collection, responses were categorized by 1 research team member according to the AAPOR standards: complete with all questions answered (AAPOR code 1.1), incomplete with primary outcome questions answered (AAPOR code 1.2), or incomplete with primary outcome questions unanswered (AAPOR code 2.12).^30^ Completed (AAPOR code 1.1) and incomplete (AAPOR code 1.2) responses that answered the primary outcome questions were included in the primary analysis. Incomplete responses with primary outcome questions unanswered (AAPOR code 2.12) were used in analyses for which there were answers provided. For each additional analysis beyond the primary outcome, we used all available responses for each question. Nonresponse was coded as AAPOR code 3.19.

### Measures

#### SGM Status

SGM status was assigned using 2 questionnaire items: (1) Do you identify as transgender? (yes, no, or prefer not to say) and (2) Which of the following best describes how you think of yourself? (bisexual, gay or lesbian, queer, straight, prefer to self-describe [free-text], or prefer not to say). These responses were dichotomized as SGM (ie, respondents identified as transgender, bisexual, gay or lesbian, queer, or an additional identity via free-text response review) and non-SGM (ie, straight, cisgender respondents). For the purposes of this analysis, non-SGM women included respondents who identified as women, not transgender, and straight; non-SGM men included respondents who identified as men, not transgender, and straight.

#### PFI and Burnout

The PFI was used to measure professional fulfilment and burnout. The PFI includes 6 items for the assessment of professional fulfilment, 4 items for the assessment of work exhaustion, and 6 items to assess interpersonal disengagement.^31^ The burnout score represents the mean of 10 work exhaustion and interpersonal disengagement items, scored on a Likert scale from 0 (not at all) to 4 (extremely), where 4 indicates the highest burnout score. The PFI assesses the degree of intrinsic positive reward the individual derives from their work, including happiness, meaningfulness, contribution, self-worth, satisfaction, and feeling in control when dealing with difficult problems at work. Items are measured on a 5-point Likert scale from 0 (not at all true) to 4 (completely true). The mean score represents the mean of all 6 items and ranges between 0 and 4. Burnout and professional fulfilment scores were rescaled to range from 0 to 10 to align with recent reports. The established thresholds (using scales ranging from 0 to 10) define 3.25 or greater as burnout and greater than 7.50 as professional fulfilment.^31^

#### Intent to Leave

Attending physicians were asked if they intended to leave their institution within 2 years (“What is the likelihood that you will leave your institution within 2 years?”) Trainees were asked if they intended to leave training (“What is the likelihood that you will leave your current training specialty within 2 years?”) The response choices were none, slight, moderate, likely, and definitely. The responses were then collapsed to form a binary variable (0 indicates none and 1, otherwise) indicating that the participants have at least a slight likelihood of leaving.

#### Anxiety and Depression

Anxiety and depression are measured using the Patient-Reported Outcomes Measurement Information System (PROMIS) short-form 4-item surveys.^32,33^ Each PROMIS scale provides a raw score ranging from 4 to 20. Raw scores are converted to T scores using the PROMIS conversion tables. T scores allow for standardized comparison across symptoms and calculation of a composite symptom score. A T score of 50 on each PROMIS symptom scale represents the general population norm, and each 10-point deviation represents 1 SD from the population norm. For the purposes of the study, a cut point of 55 or greater was used to represent a clinically elevated symptom score, as this is at least 0.5 SD worse than the population norm, which is traditionally considered a moderate effect size.

### Statistical Analysis

Analyses were completed from June 1, 2023, to February 29, 2024, using SPSS Software, version 28.0.1.1 (IBM Corporation). In alignment with the methodology of the validation study of the PFI survey instrument,^31^ and with subsequent studies using the PFI instrument, a response was defined as answering at least 75% of items within 1 or more outcome measure (ie, burnout, professional fulfillment, and intent to leave). An individual respondent’s data were included in the analyses if at least 75% of items within each survey measure were answered.

Standard descriptive summary statistics including mean (SD) were used to describe sample characteristics and prevalence of reported outcomes. We used χ^2^ tests of independence to evaluate differences by SGM status. We specified multivariable logistic regression models adjusting for age as well as self-reported race and ethnicity (categories included Asian, Hispanic of any race, non-Hispanic Black, non-Hispanic White, other [“something else” in the survey; included American Indian or Alaska Native, Native Hawaiian or Other Pacific Islander, prefer to self-describe, and multiple categories], and missing) to assess the association of SGM status with burnout, professional fulfillment, and intent to leave the institution (for attendings) or training (for trainees) as well as self-reported anxiety and depression. We elected to adjust for these demographic variables because of their known association with professional fulfillment and burnout. Additionally, research on SGM populations notes that SGM populations tend to be more racially and ethnically diverse than the general population; we wanted to ensure our study can added to this body of work.While each model 0 is unadjusted, each model 1 is adjusted for age and race and ethnicity. Each model 2 is adjusted for all demographic characteristics in model 1, as well as burnout and professional fulfillment. Statistical significance in these models was set at 2-sided *P* < .01.

## Results

Fifteen academic medical institutions conducted the Professional Well-Being Academic Consortium survey between October 1, 2019, and July 31, 2021, as described in detail elsewhere.^17^ Eight participating institutions asked sexual orientation and gender identity demographic questions of attendings, and 6 institutions asked SGM demographic questions of trainees. Surveys including SGM demographic questions were emailed to 20 541 attending-level medical specialists and 6900 trainees. Of these, 10 980 attendings and 3465 trainees completed the professional fulfillment outcome measure (>75% of items answered), resulting in an overall survey response rate of 52.6%, including 53.5% for attendings and 50.2% for trainees. After nonphysician medical specialists (dentistry, clinical psychology, podiatry, and medical physics) were removed, a total of 8376 attending physician respondents (76.3%) and 2564 trainee physician respondents (74.0%) met the analytic sample response criteria by completing at least 1 sexual orientation and gender identity question to ascertain SGM status, totaling a study population of 10 940 participants.

Respondent ages were categorized from 29 years or younger to 60 years of older. The race and ethnicity of respondents included Asian (1698 [15.5%]), Hispanic of any race (501 [4.6%]), non-Hispanic Black (268 [2.4%]), non-Hispanic White (7304 [66.8%]), other (including American Indian or Alaska Native, Native Hawaiian or Other Pacific Islander, prefer to self-describe, and multiple categories; 771 [7.0%]), and missing (398 [3.6%]). Additional details about the personal and professional characteristics of responders, including number of respondents, age bracket, and clinician type (attending and trainee), are shown in Table 1. Of 10 940 total respondents who provided SGM status, 386 of 8376 attendings (4.6%) and 212 of 2564 trainees (8.3%) identified as SGM.

**Table 1.** Demographic Characteristics of Survey Respondents by SGM Status^a^

Characteristic	Attendings			Trainees		
	No. (%) of respondents		*P* value	No. (%) of respondents		*P* value
	SGM status (n = 386)	Non-SGM status (n = 7990)		SGM status (n = 212)	Non-SGM status (n = 2352)	
Age category, y						
≤29	<5	23 (0.3)	<.001	80 (37.7)	844 (35.9)	.79
30-39	136 (35.2)	2016 (25.2)		127 (59.9)	1458 (62.0)	
40-49	118 (30.6)	2583 (32.3)		<5	36 (1.5)	
50-59	86 (22.3)	1860 (23.3)		<5	<5	
≥60	41 (10.6)	1456 (18.2)		<5	<5	
Missing	<5	52 (0.7)		<5	11 (0.5)	
Race and ethnicity						
Asian	34 (8.8)	1132 (14.2)	<.001	35 (16.5)	497 (21.1)	.22
Hispanic, any race	36 (9.3)	300 (3.8)		14 (6.6)	151 (6.4)	
Non-Hispanic Black	16 (4.1)	163 (2.0)		12 (5.7)	77 (3.3)	
Non-Hispanic White	266 (68.9)	5545 (69.4)		131 (61.8)	1362 (57.9)	
Other^b^	25 (6.5)	574 (7.2)		13 (6.1)	159 (6.8)	
Missing	9 (2.3)	276 (3.5)		7 (3.3)	106 (4.5)	

Abbreviation: SGM, sexual and gender minority.

^a^ Percentages have been rounded and may not total 100. Cells with fewer than 5 participants are excluded for confidentiality purposes.

^b^ Called “something else” in the survey and included American Indian or Alaska Native, Native Hawaiian or Other Pacific Islander, prefer to self-describe, and multiple categories.

### Burnout

Compared with their non-SGM peers, prevalence of burnout was higher among SGM attendings (181 of 382 [47.4%] vs 2791 of 7883 [35.4%]; *P* < .001) and trainees (108 of 211 [51.2%] vs 954 of 2332 [40.9%]; *P* < .001) (Table 2). After adjusting for age and race and ethnicity, SGM attendings had increased odds of reporting burnout compared with their non-SGM peers (adjusted odds ratio [AOR], 1.57 [95% CI, 1.27-1.94]; *P* < .001) (Table 3). Similar results were found among SGM trainees (AOR, 1.47 [95% CI, 1.10-1.96]; *P* = .01) (Table 4).

**Table 2.** Outcomes of Interest by SGM Status

Outcome	Attendings					Trainees				
	SGM status		Non-SGM status		*P* value	SGM status		Non-SGM status		*P* value
	Total No. of responses	No. (%) with outcome	Total No. of responses	No. (%) with outcome		Total No. of responses	No. (%) with outcome	Total No. of responses	No. (%) with outcome	
Overall burnout^a^	382	181 (47.4)	7883	2791 (35.4)	<.001	211	108 (51.2)	2332	954 (40.9)	<.001
Professional fulfillment^a^	386	133 (34.5)	7922	3200 (40.4)	<.001	211	63 (29.9)	2333	833 (35.7)	<.001
Intent to leave	376	125 (33.2)	7873	2433 (30.9)	<.001	49	8 (16.3)	342	54 (15.8)	.22
Anxiety^a^	140	54 (38.6)	1991	514 (25.8)	<.001	207	88 (42.5)	2326	805 (34.6)	<.001
Depression^a^	133	48 (36.1)	1915	475 (24.8)	<.001	205	88 (42.9)	2323	748 (32.2)	<.001

Abbreviation: SGM, sexual and gender minority.

^a^ Using 75% inclusion criteria.

**Table 3.** Association of SGM Status With Burnout, Professional Fulfillment, Intent to Leave, Anxiety, and Depression Among Attending Survey Respondents

Dependent variable	Model^a^	Odds ratio (95% CI)	*P* value
Burnout	0	1.64 (1.34-2.02)	<.001
	1	1.57 (1.27-1.94)	<.001
	2a	1.53 (1.20-1.95)	<.001
Professional fulfillment	0	0.78 (0.63-0.96)	.02
	1	0.80 (0.64-0.99)	.04
	2b	0.97 (0.76-1.24)	.81
Intent to leave	0	1.11 (0.89-1.39)	.34
	1	1.12 (0.89-1.40)	.35
	2	0.98 (0.77-1.24)	.86
Anxiety	0	1.79 (1.44-2.23)	<.001
	1	1.68 (1.34-2.10)	<.001
	2	1.51 (1.19-1.93)	<.001
Depression	0	1.74 (1.40-2.17)	<.001
	1	1.72 (1.37-2.15)	<.001
	2	1.53 (1.18-1.97)	.001

Abbreviation: SGM, sexual and gender minority.

^a^ Zero indicates unadjusted; 1, adjusted for age and race and ethnicity; 2, adjusted for age, race and ethnicity, burnout, and personal fulfillment; 2a, adjusted for age, race and ethnicity, and personal fulfillment; and 2b, adjusted for age, race and ethnicity, and burnout.

**Table 4.** Association of SGM Status with Burnout, Professional Fulfillment, Intent to Leave, Anxiety, and Depression Among Trainee Survey Respondents

Dependent variable	Model^a^	Odds ratio (95% CI)	*P* value
Burnout	0	1.52 (1.14-2.01)	.004
	1	1.47 (1.10-1.96)	.01
	2a	1.47 (1.05-2.04)	.03
Professional fulfillment	0	0.77 (0.57-1.04)	.09
	1	0.80 (0.59-1.09)	.16
	2b	0.96 (0.67-1.37)	.82
Intent to leave	0	1.04 (0.46-2.34)	.92
	1	1.03 (0.43-2.47)	.95
	2	1.02 (0.42-2.47)	.96
Anxiety	0	1.40 (1.05-1.86)	.02
	1	1.37 (1.02-1.84)	.04
	2	1.23 (0.89-1.70)	.21
Depression	0	1.58 (1.18-2.11)	.002
	1	1.60 (1.19-2.16)	.002
	2	1.48 (1.05-2.09)	.02

Abbreviation: SGM, sexual and gender minority.

^a^ Zero indicates unadjusted; 1, adjusted for age and race and ethnicity; 2, adjusted for age, race and ethnicity, burnout, and personal fulfillment; 2a, adjusted for age, race and ethnicity, and personal fulfillment; and 2b, adjusted for age, race and ethnicity, and burnout.

### Professional Fulfillment

Compared with their non-SGM peers, levels of professional fulfillment were lower among SGM attendings (133 of 386 [34.5%] vs 3200 of 7922 [40.4%]; *P* < .001) and trainees (63 of 211 [29.9%] vs 833 of 2333 [35.7%]; *P* < .001) (Table 2). After adjusting for age and race and ethnicity, SGM attendings had lower odds of reporting professional fulfillment compared with their non-SGM peers (AOR, 0.80 [95% CI, 0.64-0.99]; *P* = .04), but these results were not statistically significant at the *P* < .01 threshold (Table 3). There was no difference among SGM trainees compared with their non-SGM peers when adjusting for age and race and ethnicity (AOR, 0.80 [95% CI, 0.59-1.09]; *P* = .16) (Table 4).

### Intent to Leave

Intent to leave institution was higher among SGM attendings than their non-SGM peers (125 of 376 [33.2%] vs 2433 of 7873 [30.9%]; *P* < .001) (Table 2). There was no difference in rates of intent to leave training among trainees by SGM status. After adjusting for age and race and ethnicity, SGM attendings were no more or less likely to express an intent to leave than non-SGM peers (AOR, 1.12 [95% CI, 0.89-1.40]; *P* = .35) (Table 3). Findings were similar for SGM and non-SGM trainees (AOR, 1.03 [95% CI, 0.43-2.47]; *P* = .95) (Table 4).

### Anxiety and Depression

Compared with their non-SGM peers, prevalence of self-reported anxiety was higher among SGM attendings (54 of 140 [38.6%] vs 514 of 1991 [25.8%]; *P* < .001) and trainees (88 of 207 [42.5%] vs 805 of 2326 [34.6%]; *P* < .001) (Table 2). Similar results were found for depression (48 of 133 [36.1%] vs 475 of 1915 [24.8%] among attendings and 88 of 205 [42.9%] vs 748 of 2323 [32.2%] among trainees; *P* < .001 for both). After adjusting for age and race and ethnicity, SGM attendings had increased odds of reporting anxiety (AOR, 1.68 [95% CI, 1.34-2.10]; *P* < .001) compared with non-SGM peers; similar results were found for self-reported depression (AOR, 1.72 [95% CI, 1.37-2.15]; *P* < .001). These findings persisted even when adjusting for burnout and professional fulfillment (Table 3).

Similar results were found among SGM trainees compared with their non-SGM peers. After adjustment, SGM trainees had an increased likelihood of self-reporting depression (AOR, 1.60 [95% CI, 1.19-2.16]; *P* = .002). This difference was not significant when further adjusting for burnout and professional fulfillment (Table 4).

## Discussion

This cross-sectional survey study is the first, to our knowledge, to report the prevalence of SGM status in a large, multicenter cohort of physicians, as well as to report the association of SGM status with worse occupational well-being. In our study, 4.6% of attendings and 8.3% of trainees identified as having SGM status. Our study found significant differences in well-being by SGM status. Even when adjusting for age and race and ethnicity, SGM attendings had increased odds of burnout and decreased odds of professional fulfillment. Similarly, SGM trainees had increased odds of burnout.

The prevalence of SGM status among clinicians has not been well studied. While persons with SGM status have been part of the profession since its outset, their exact number and experience remain largely unknown. This is due, in part, to the stigmatization and historical pathologization of SGM identity.^34,35^ To begin to address the lack of research on SGM physicians and trainees, the Association of American Medical Colleges includes sexual orientation and gender identity questions in their surveys of matriculating and graduating medical students.^36,37^ In 2016, medical school graduates who identified as gay or lesbian constituted 3.1% of respondents, with 2.1% identifying as bisexual^37^; this increased to 4.1% and 5.3%, respectively, in 2023.^36^ Medical graduates who reported a different gender identity from their sex assigned at birth increased from 1.1% in 2022 to 1.3% in 2023.^36^ Similar data are lacking for physicians who have completed training; the American Medical Association began allowing members to voluntarily report their SGM status, but these data are not publicly available.^38^

The prevalence of self-reported SGM status within the physician workforce is important in the context of the patient population we serve. Approximately 7.6% of all adults in the US and 22.3% of persons born between 1997 and 2012 (Generation Z) identify as SGM.^39^ Recognizing and responding to these evolving demographic trends in our patients is a fundamental principle of medicine, essential to maintaining professional excellence, fulfilling the promise of precision medicine, and meeting our patients’ unique needs.^40^ Our findings suggest that SGM individuals are underrepresented among attending clinicians relative to the general population and are likely to continue to be underrepresented as the population ages. Evidence suggests that current medical education does not fully equip physicians with the knowledge and skills needed to provide high-quality care for SGM patients.^41,42^ There is a need for physicians and trainees who understand the unique needs of SGM patients and communities, both through training^28^ as well as through personal experience as an SGM person.

Given the importance of SGM physicians in the health care workforce, we must understand the factors that affect their recruitment and retention. Prior to our study, little was known about the occupational well-being of SGM physicians. The few studies examining the experiences of SGM physicians^19,43,44,45,46,47,48^ are limited in scope and generalizability, but all consistently found that SGM physicians face considerable challenges in the workplace, and that most of their non-SGM colleagues have had little to no training on SGM issues. Similarly, LGB medical students were more likely to have less favorable perceptions of their learning environment compared with their non-LGB peers.^49^

We found that SGM physicians and trainees report higher rates of burnout and lower rates of professional fulfillment than their non-SGM peers. To our knowledge, no previous study has explored the differential drivers of occupational well-being for SGM physicians and trainees. We postulate that several factors may account for our findings, including social stigma, reduced sense of belonging, experiences of mistreatment, and lack of mentorship and sponsorship. In support of this hypothesis, our findings align with existing data suggesting that being forced to hide lived experience or to assimilate via passing (ie, hiding SGM status) or covering (ie, minimizing SGM status) can have a negative effect on well-being and performance.^50,51^ These burdens to assimilate likely contribute to burnout and increased risk of leaving an institution and potentially the profession.

Our study found that SGM status was associated with increased intent of attendings to leave their current institution, but not for trainees. This may be due to student loan debt and other financial pressures that contribute to decision-making about completing training. Given the increased prevalence of SGM status among trainees relative to attendings, it may be that there is a greater sense of community for SGM trainees than for their attending counterparts. Further research is needed to better understand this difference.

An understanding of the occupational well-being of physicians is incomplete if it does not include SGM status. Making the experience of SGM physicians and trainees visible will allow interventions to be deployed that improve their well-being and retention. With the inclusion of SGM identity in surveys of physicians and trainees, coupled with more comprehensive and robust data collection about physicians’ identities, social positions, and processes of oppression and privilege, researchers would be able to conduct more thoughtful intersectional analyses using large datasets, thereby identifying intervenable drivers of burnout and fulfillment among SGM physicians, including those with multiple minority identities. Additionally, with more surveys inclusive of SGM status, specialty-specific issues could be explored.

### Limitations

Our study has several limitations. First, as a cross-sectional analysis, we cannot specifically comment on causation between SGM identity and workplace experience. However, repeated research in different settings continues to find consistent disparities in workplace experiences by SGM status. Second, the sample size of this study precludes an analysis of the interplay between multiple identities (ie, gender and race and ethnicity) and similarly precludes adjusting for specialty. Third, while the overall response rate of 52.6% was high for physician surveys, fewer than half of potential respondents both completed the survey and provided data on at least 1 element of SGM status. Not all respondents provided complete data on each aspect of SGM status, leaving open the possibility that some SGM respondents were counted among the non-SGM population. Thus, there remains the possibility of significant response bias. Last, the etiology of rate of missingness of SGM status is unknown in the present study due to lack of data on SGM status for the population of all survey invitees. The rate of SGM status may be underreported or overreported. Future studies exploring reticence or willingness to report SGM status by jurisdiction (eg, antidiscrimination policies) and hospital ownership (eg, religious affiliation) may prove telling.

## Conclusions

In a national cohort of physicians, SGM attendings and trainees demonstrated higher levels of burnout and lower levels of professional fulfillment. SGM attendings reported a higher intent to leave their institution than non-SGM peers, but SGM trainees did not. These findings warrant further research, which will allow for the development of targeted interventions to retain a health care workforce that can meet our patients’ needs.

## Notes

Supplement.
Data Sharing Statement
