# Supplementary material for: Academic Physician and Trainee Occupational Well-Being by Sexual and Gender Minority Status
Source: JAMA Netw Open. 2024 Nov 13;7(11):e2443937. doi: 10.1001/jamanetworkopen.2024.43937 (PMC11561694; doi:10.1001/jamanetworkopen.2024.43937)
Supplement: Supplement. — Data Sharing Statement [file jamanetwopen-e2443937-s001.pdf]

Data Sharing Statement

Streed, Jr. Academic Physician and Trainee Occupational Well-Being by Sexual and Gender Minority Status. *JAMA Netw Open*. Published November 13, 2024.
doi:10.1001/jamanetworkopen.2024.43937

Data
Data available: No

Additional Information
Explanation for why data not available: Data requst can be made to the healthcare professional well-being academic consortium (https://healthcarepwac.org/).
